# An automated CPR device compared with standard chest compressions for out-of-hospital resuscitation

**DOI:** 10.1186/1471-227X-12-8

**Published:** 2012-06-26

**Authors:** Paul A Jennings, Linton Harriss, Stephen Bernard, Janet Bray, Tony Walker, Tim Spelman, Karen Smith, Peter Cameron

**Affiliations:** 1 Ambulance Victoria, Manningham Road, Doncaster, Australia; 2 Department of Epidemiology and Preventive Medicine, Monash University, Commercial Road, Melbourne, Australia; 3 National Stroke Foundation, Bourke Street, Melbourne, Australia

## Abstract

**Background:**

Effective cardiopulmonary resuscitation and increased coronary perfusion pressures have been linked to improved survival from cardiac arrest. This study aimed to compare the rates of survival between conventional cardiopulmonary resuscitation (C-CPR) and automated CPR (A-CPR) using AutoPulse™ in adults following out-of-hospital cardiac arrest (OHCA).

**Methods:**

This was a retrospective study using a matched case–control design across three regional study sites in Victoria, Australia. Each case was matched to at least two (maximum four) controls using age, gender, response time, presenting cardiac rhythm and bystander CPR, and analysed using conditional fixed-effects logistic regression.

**Results:**

During the period 1 October 2006 to 30 April 2010 there were 66 OHCA cases using A-CPR. These were matched to 220 cases of OHCA involving the administration of C-CPR only (controls). Survival to hospital was achieved in 26% (17/66) of cases receiving A-CPR compared with 20% (43/220) of controls receiving C-CPR and the propensity score adjusted odds ratio [AOR (95% CI)] was 1.69 (0.79, 3.63). Results were similar using only bystander witnessed OHCA cases with presumed cardiac aetiology. Survival to hospital was achieved for 29% (14/48) of cases receiving A-CPR compared with 18% (21/116) of those receiving C-CPR [AOR = 1.80 (0.78, 4.11)].

**Conclusions:**

The use of A-CPR resulted in a higher rate of survival to hospital compared with C-CPR, yet a tendency for a lower rate of survival to hospital discharge, however these associations did not reach statistical significance. Further research is warranted which is prospective in nature, involves randomisation and larger number of cases to investigate potential sub-group benefits of A-CPR including survival to hospital discharge.

## Background

The relationship between effective cardiopulmonary resuscitation (CPR) and improved survival of patients suffering cardiac arrest is clear [1]. Unfortunately, the quality of CPR performed by health care professionals in both the in-hospital and out-of-hospital environments is often poor [2,3]. Examination of the CPR practices of health care professionals in both of these environments reveals that chest compressions are too few and shallow, too many ventilations are given, and there are significant pauses during active chest compressions [2,3]. Each of these errors may significantly reduce the chance of successful resuscitation.

The use of a mechanical automated chest compression device (A-CPR), may lead to superior coronary perfusion pressures by addressing the shortcomings of conventional manual CPR (C-CPR) [4], thus improving survival rates from out-of-hospital cardiac arrest (OHCA). The 2010 European Resuscitation Council Guidelines suggest that mechanical devices may have an important role in the resuscitation of patients in the prehospital environment [5]. Studies investigating the use of this device are limited. Laboratory and clinical studies have shown blood pressure levels approaching normal levels with automatic chest compression devices and better neurological outcomes following prolonged cardiac arrest [6-8]. Three human studies to date have shown a similar effect on coronary perfusion pressures and also improved rates of return of spontaneous circulation (ROSC), [9-11] but conflicting effects on survival to hospital discharge.

Ambulance Victoria introduced seven A-CPR units across three mixed urban and rural areas in 2006 to support paramedics in the provision of external chest compressions, particularly in the rural areas where outcomes had been shown to be poorer [12]. It was proposed that the A-CPR unit would assist during CPR because the number of paramedics at the scene at rural cardiac arrest is often less than metropolitan areas [12].

This study was undertaken to compare the rates of survival to hospital between C-CPR and A-CPR in adults following OHCA in this setting.

## Methods

### Study design

This study used a matched case–control method (1 case: 4 controls where available [min 2, max 4 controls]) [13] using prospectively collected case data matched to Victorian Ambulance Cardiac Arrest Registry (VACAR) data. The VACAR database contains case data on all OHCA attended by Emergency Medical Services (EMS) in the state of Victoria, Australia. All adult (>18 years of age) OHCA cases using the A-CPR (AutoPulse®, Zoll Medical Corporation, Chelmsford, MA, USA) were matched to cases receiving C-CPR. All cases were matched by known predictors of survival [14]; age (+/− 5 years), gender, response time (defined as ‘at patient’ – ‘call received’ time,+/− 5 minutes), presenting cardiac rhythm (VF / VT / PEA / Asystole), and the presence of bystander CPR. Paramedics were trained to commence manual chest compressions whilst setting up the A-CPR device and to apply the device with minimal interruption to chest compressions. All controls were selected from regional settings similar to those of the A-CPR trial sites. The primary outcome measure was survival to hospital (defined as pulse on arrival to hospital in the absence of chest compressions). The Monash University Human Research Ethics Committee approved the study.

### Setting

The A-CPR was introduced into three mixed urban / rural settings of Ambulance Victoria. The three settings were the provincial city of Geelong (population 208,139), and the townships of Shepparton (population 58,870) and Mildura (population 45,703). The regions employ a two-tier response system comprising Advanced Life Support (ALS) paramedics who have a range of advanced life support skills (laryngeal mask airway, intravenous adrenaline, intravenous fluids) and Mobile Intensive Care Ambulance (MICA) paramedics who are authorised to perform endotracheal intubation and administer a range of cardiac drugs, including adrenaline, amiodarone and atropine. (see http://www.ambulance.vic.gov.au)

The responding skill set is determined by a computerised call taking and dispatch system (Advanced Medical Priority Dispatch System, Salt Lake City, Utah), and dispatches the closest and most appropriate resource based on the nature of the case. A-CPR devices were placed on ambulance vehicles staffed by ALS paramedics, MICA paramedics, or mixed ALS/MICA paramedic crews as these vehicles were more likely to arrive first at scene.

### Statistical analysis

Continuous data was reported as medians (IQR) due to non-parametric distribution, and frequencies are expressed as percentages. Adjusted odds ratios (ORs) were calculated using conditional logistic regression with C-CPR cases as the reference group and controlling for confounders previously described (age, gender, response time, rhythm on arrival, bystander CPR). Confidence limits were set at the 95% level and two-sided *P* values are presented. We have attempted to deal with potential selection bias introduced via the non-random assignment of treatment groups, in part, by correcting through the derivation of propensity scores as an adjunct to the matching already described. Deriving and adjusting for propensity score aims to reduce such bias in estimating the treatment effect in non-randomised observational studies [15]. A subgroup analysis was undertaken for bystander witnessed OHCA with presumed cardiac aetiology. Too few cases involved survival to hospital discharge to consider this as a legitimate outcome. All reported p-values were two-tailed and for each analysis p < 0.05 was considered significant. All statistical analyses were performed using Stata 11 (StataCorp. Stata Statistical Software: Release 11. In. College Station, TX: StataCorp LP; 2009).

## Results

During the period October 2006 to April 2010 there were 66 OHCAs where A-CPR was administered, and these were matched to 220 controls (mean 3.3 controls per A-CPR case) selected from 1,610 cardiac arrests which occurred during the study period (Table 1). Table 2 summarises the characteristics of the A-CPR and C-CPR groups. The median time to application of A-CPR from arrival was 4 minutes (IQR 2–7 mins). Survival to hospital was achieved in 26% (17/66) of OHCAs receiving A-CPR compared with 20% (43/220) for those receiving C-CPR, however this finding was not statistically significant. Cases receiving A-CPR were 70 percent more likely to survive to hospital than those receiving C-CPR [AOR = 1.69 (0.79, 3.63)], but again this finding was not statistically significant.

**Table 1:** Characteristics of the entire cohort (n = 1,610) who were eligible for matching and received C-CPR

**Characteristics**		**C-CPR**
		**n = 1,610**
Age median [IQR](years)		67 [54–78]
Gender Male n(%)		1,124 (70)
Bystander CPR n(%)		689 (43)
Initial Rhythm		
	Asystole n(%)	605 (38)
	VF/VT n(%)	450 (28)
	PEA n(%)	239 (15)
	Other n(%)	306 (19)
	Unknown n(%)	10 (1)
Witnessed status		
	Witnessed n(%)	808 (50)
	Witnessed by EMS n(%)	270 (17)
	Not witnessed n(%)	519 (32)
Precipitating Event		
	Presumed cardiac n(%)	1,299 (81)
	Respiratory n(%)	75 (5)
	Neurological n(%)	23 (1)
	Overdose n(%)	36 (2)
	Hanging n(%)	19 (1)
EMS Response time median [IQR]		10 [7-16]
Overall survival to hospital n(%)		349 (22)
Overall survival to hospital discharge n(%)*		109 (7)

IQR: Inter-quartile range; *Data missing for 85 cases.

**Table 2:** Characteristics of cases and controls

**Characteristics**		**A-CPR**	**C-CPR**	**P Value**
		**n = 66**	**n = 220**	
Age median [IQR](years)		69 [53–78]	71 [55–77.5]	0.53*
Gender Male n(%)		36 (55)	135 (61)	0.32^#^
Bystander CPR n(%)		33 (54)	127 (62)	0.25^#^
Initial Rhythm				
	Asystole n(%)	29 (44)	88 (40)	0.50^
	VF/VT n(%)	20 (30)	78 (36)	
	PEA n(%)	8 (12)	28 (13)	
	Other n(%)	8 (12)	26 (11)	
	unknown n(%)	1 (2)	0	
Witnessed status				
	Witnessed n(%)	39 (59)	133 (60)	0.98^#^
	Witnessed by EMS n(%)	8 (12)	26 (12)	
	Not witnessed n(%)	19 (29)	61 (28)	
Precipitating Event				
	Presumed cardiac n(%)	57 (86)	211 (96)	0.02^
	Respiratory n(%)	5 (7)	6 (3)	
	Neurological n(%)	1 (2)	0	
	Overdose n(%)	2 (3)	3 (1)	
	Hanging n(%)	1 (2)	0	
EMS Response time median [IQR]		9 [7-13]	9 [7-13]	0.73*
Time from arrival to A-CPR activation		4 [2-7]	NA	
Overall survival to hospital n(%)		17 (26)	43 (20)	0.23^#^
Overall survival to hospital discharge n(%)		2 (3)	15 (7)	0.38^

IQR: Inter-quartile range; NA: Not Applicable *Mann–Whitney test; ^#^Chi Squared test; ^Fischer’s exact test.

Few cases of OHCA survived to hospital discharge from either group; three percent (2/66) of those receiving A-CPR compared with 7% (15/220) or those receiving C-CPR (p = 0.38).

For sub-group analysis, we included only bystander witnessed, presumed cardiac aetiology OHCAs. Survival to hospital was achieved in 29% (14/48) of people receiving A-CPR compared with 18% (21/116) of those receiving C-CPR. Cases receiving A-CPR were eighty percent more likely to survive to hospital compared with cases receiving C-CPR [AOR = 1.80 (0.78, 4.11)], although again this difference was not statistically significant.

Table 3 describes the outcomes categorised by shockable or non-shockable rhythm on arrival of the EMS. The largest proportion of survivors to hospital arose from the A-CPR group who presented with a shockable rhythm.

**Table 3:** Outcomes by initial, presenting rhythm

**Initial Rhythm**	**Survived to hospital (n = 57**^**#**^**)**			**Survived to hospital discharge (n = 14**^**#**^**)**		
	**No./Total No. Of Patients (%)**			**No./Total No. Of Patients (%)**		
	**A-CPR**	**C-CPR**	P Value*	**A-CPR**	**C-CPR**	P Value*
Shockable rhythm (VF/VT)	10/20 (50)	26/78 (33)	0.20	0/20 (0)	7/78 (9)	0.34
Non-shockable rhythm (Asystole/ PEA)	7/37 (19)	14/116 (12)	0.28	1/37 (3)	6/116 (5)	1.00

^#^ Excludes cases where initial rhythm was ‘unknown’ or ‘other’ *Fisher’s exact test.

## Discussion

The use of A-CPR resulted in a higher rate of survival to hospital compared with C-CPR, yet a tendency for a lower rate of survival to hospital discharge, however these associations did not reach statistical significance. We matched cases to controls using important predictors of survival (age, gender, response time, presenting cardiac rhythm, bystander CPR and regional setting), and adjusted for potential confounding through conditional multiple regression techniques and adjusting for propensity score. The matching process appeared effective as there was little change in the estimate of association when the propensity score was added to the univariable regression model.

Our findings are consistent with a number of other prehospital studies comparing A-CPR to conventional resuscitation [10,11,16,17]. Ong et al compared manual compressions (N = 499) to A-CPR compressions (N = 284) in OHCA patients and found an improved rate of ROSC (34.5% v 20.2%; AOR = 1.94, 95% CI 1.38-2.72), survival to hospital admission (20.9% v 11.1%; AOR = 1.88, 95% CI 1.23-2.86) and survival to hospital discharge (9.7% v 2.9%; OR = 2.27, 95% CI 1.11-4.77) [10]. In a case–control study reported by Casner et al, the proportion of patients achieving sustained ROSC was also found to be greater in the A-CPR group than the C-CPR group (39% v 29%; p = 0.003) [17]. This study also found that more patients presenting in asystole or agonal rhythms had a sustained ROSC with A-CPR. These findings are consistent with our study. A study by Krep et al found the AutoPulse system to an effective and safe mechanical CPR device and useful in the management of out-of-hospital cardiac arrest [18].

However, a third study did not find improvement in outcome above C-CPR. Hallstrom et al conducted a large, multicentre randomised controlled trial comparing C-CPR to A-CPR. They reported similar proportions of patients surviving to ED (C-CPR 41.3% v A-CPR 40.4%) but a lower proportion of A-CPR being discharged from hospital alive (9.9% v 5.8%; OR = 0.56; P = 0.06) [11].

The current European Resuscitation Council Guidelines [5] identify that clinical trials investigating the role of mechanical devices to date have been conflicting. They conclude that mechanical devices have been used effectively to support patients in special circumstances (i.e. undergoing primary coronary intervention and CT scans, and also for prolonged resuscitation attempts) where rescuer fatigue may impair the effectiveness of manual chest compression. Whilst cautioning that the role of mechanical devices still require further evaluation, they acknowledge that mechanical devices may also have a role in the prehospital environment where extrication of patients, resuscitation in confined spaces and movement of patients on a trolley often preclude effective manual chest compressions [5].

Several studies have shown that survival from OHCA is much lower in rural areas than urban areas [12,19]. One study showed Urban patients with bystander-witnessed cardiac arrest were more likely to arrive at an emergency department with a cardiac output (odds ratio [OR], 2.92; 95% CI, 1.65–5.17; P < 0.001), and to be discharged from hospital alive than rural patients (urban, 125/1685 [7.4%]; rural, 2/105 [1.9%]; OR, 4.13; 95% CI, 1.09–34.91) [12]. This disparity is often thought to be solely as a result of longer travel distances and time between collapse and defibrillation, but it is likely to be multifactorial. Often there are fewer prehospital clinicians attending a rural cardiac arrest, compared to urban cardiac arrests, which limit the number of interventions which can be performed concurrently whilst maintaining consistent, high quality chest compressions.

The use of A-CPR has several potential advantages in a rural setting. Chest compressions are able to be provided effectively in the back of a moving vehicle en route to hospital. Without such a device, paramedics are unrestrained and are at risk of injury in a moving vehicle. Furthermore, mechanical devices do not tire, and maintain consistent depth and rate of compressions.

The main disadvantage of A-CPR is the substantial weight of the device (11.6 kg including battery).

### Limitations

This study was potentially limited by the low number of patients enrolled in the A-CPR arm during the study period. Also, treatment was not randomised in this study, however we attempted to minimize bias using a matched case–control design and by the use of propensity scores to adjust for known and unknown confounding factors.

Finally, survival rates are lower in rural areas when compared to urban centres [12], making it difficult to recruit sufficient numbers to detect a difference in outcome and therefore evaluate the true utility of A-CPR in the rural and regional prehospital setting.

## Conclusions

A-CPR may improve rate of survival to hospital over traditional C-CPR in selected settings and warrant further studies of this device, particularly examining the potential utility in rural settings.

## Competing interests

Zoll Medical Australia Pty Ltd provided an unrestricted grant. The funding source had no role in the study design, data collection, data analysis, data interpretation, writing of the report or the decision to submit for publication.

## Authors’ contributions

PAJ and TS analysed the data for the present paper. PJ wrote the initial draft of the manuscript. All authors contributed to study design, interpretation of the data, intellectual discussion and revision of the manuscript. All authors have made substantive contributions to the study, and all authors endorse the data and conclusions. All authors read and approved the final manuscript.

## Pre-publication history

The pre-publication history for this paper can be accessed here:

http://www.biomedcentral.com/1471-227X/12/8/prepub
